# What counts? Adding nuance to retail food environment measurement tools in a Canadian context

**DOI:** 10.1017/S1368980023000733

**Published:** 2023-07

**Authors:** Alexa Rae Ferdinands, Jennifer Ann Brown, Charlene C Nielsen, Candace IJ Nykiforuk, Kim D Raine

**Affiliations:** School of Public Health; 3-300 Edmonton Clinic Health Academy, 11405 – 87 Ave, University of Alberta, Edmonton T6G 1C9, AB, Canada

**Keywords:** mRFEI, GIS, Food environment, Food retailer, Canada, Nutrition, Measurement, Public health

## Abstract

**Objective:**

Limitations of traditional geospatial measures, like the modified Retail Food Environment Index (mRFEI), are well documented. In response, we aimed to: (1) extend existing food environment measures by inductively developing subcategories to increase the granularity of healthy *v*. less healthy food retailers; (2) establish replicable coding processes and procedures; and (3) demonstrate how a food retailer codebook and database can be used in healthy public policy advocacy.

**Design:**

We expanded the mRFEI measure such that ‘healthy’ food retailers included grocery stores, supermarkets, hypermarkets, wholesalers, bulk food stores, produce outlets, butchers, delis, fish and seafood shops, juice/smoothie bars, and fresh and healthy quick-service retailers; and ‘less healthy’ food retailers included fast-food restaurants, convenience stores, coffee shops, dollar stores, pharmacies, bubble tea restaurants, candy stores, frozen dessert restaurants, bakeries, and food trucks. Based on 2021 government food premise licences, we used geographic information systems software to evaluate spatial accessibility of healthy and less healthy food retailers across census tracts and in proximity to schools, calculating differences between the traditional *v*. expanded mRFEI.

**Setting:**

Calgary and Edmonton, Canada.

**Participants:**

N/A.

**Results:**

Of the 10 828 food retailers geocoded, 26 % were included using traditional mRFEI measures, while 53 % were included using our expanded categorisation. Changes in mean mRFEI across census tracts were minimal, but the healthfulness of food environments surrounding schools significantly decreased.

**Conclusions:**

Overall, we show how our mRFEI adaptation, and transparent reporting on its use, can promote more nuanced and comprehensive food environment assessments to better support local research, policy and practice innovations.

## Retail food environments and public health

Poor diet quality is the second greatest risk factor for premature death and disability combined in Canada^(1)^. A poor diet is characterised by an eating pattern low in fibre, protein, and micronutrients, and high in total energy, free sugars, saturated fats, and sodium. Although Canada’s Food Guide recommends Canadians limit their intake of highly processed foods and drinks typical of a poor diet, approximately half of total daily energy intake for Canada’s population in 2015 came from these sources^(2)^.

Evidence suggests that retail food environments (RFE) (i.e. ‘the availability and composition (e.g. relative mix) of food retailing within local environments’^(3)^ ^(p. 1)^) may be an important determinant of population-level eating patterns and diet-related chronic diseases^(4,5)^. As such, improving the healthfulness of RFE has become an increasing public health concern across Canada^(6)^. In 2014, our team developed Alberta’s Nutrition Report Card on Food Environments for Children and Youth (hereafter, ‘Nutrition Report Card’) to benchmark policies and practices that support or inhibit healthy eating practices in the Canadian province of Alberta^(7)^. Two Nutrition Report Card indicators targeted RFE, acknowledging that the relative mix, or ratio, of healthy to less healthy food retailers (henceforth, ‘retailers’) in a defined geographical area can impact people’s food purchasing and eating practices^(8)^. Indicators evaluating spatial accessibility of healthy and less healthy retailers within the RFE included: 1) availability of healthy retailers across census tracts in municipalities of interest; and 2) availability of less healthy retailers in proximity to schools^(7)^. To categorise retailers as ‘healthy’ and ‘less healthy’, we used the Centers for Disease Control and Prevention’s (CDC)^(9)^ modified Retail Food Environment Index (mRFEI) (see section 2·2).

## Challenges in measuring retail food environments

While calculating the mRFEI represents a common approach to assess relative density of healthy retailers^(9,10)^, it is limited in detail and differentiation^(11,12)^. Examples of these limitations are outlined here:

- Narrow view of the RFE. Calculating the proportion of healthy to less healthy retailers from the CDC’s list of six retailer types (see section 2.2) meant excluding retailers that potentially shape purchasing and eating practices, like coffee shops and pharmacies^(13,14)^. Further, given the heterogeneity of food and beverage offerings and/or location characteristics, it was often challenging to determine whether to include or exclude certain retailers. For example, were fish and seafood stores included under the supermarkets and other grocery stores category, or excluded?
- Imprecision within retailer categories. Vague categories restricted our ability to infer details about retailer types. For instance, within the limited-service eating places category, we could not distinguish between less healthy fast-food restaurants and potentially healthy sandwich shops. In another example, ethnic food stores typically sell many convenience and pre-packaged foods, leading them to be categorised as less healthy convenience stores, despite also offering healthy (and affordable) culturally specific foods and beverages^(15,16)^. Thus, mRFEI categorisations may misrepresent the role of ethnic grocers as healthy options. Greater precision (i.e. capturing subcategories of retailers) could offer valuable details for stakeholders like public health practitioners, policymakers and urban planners in identifying opportunities to improve RFE healthfulness.
- Shortcomings of simplistic binary labelling systems. The mRFEI only categorises retailers by type, without accounting for the range of foods sold within^(17)^. For example, a grocery store may sell an abundance of highly processed foods but remains labelled as ‘healthy’. Additionally, RFE are dynamic, continuously adapting to new business practices. Fast-food restaurants have gradually increased their healthy offerings, which can be problematic when applying binary healthfulness labels^(10)^.
- Potential for inconsistencies and misclassifications. Despite using codebooks and outlining procedures to promote inter-coder reliability, coder subjectivity can still result in inconsistencies in determining whether to include/exclude retailers, and in categorising included retailers^(18)^.

Researchers have documented concerns about the reliability of RFE measurement approaches across coders and contexts for years^(8,12,13,18–20)^. Wilkins *et al.* called for transparent reporting of geospatial RFE analysis, arguing that more attention to how retailer types are defined, and reaching consensus on definitions, would enhance comparability across studies^(18)^. Han *et al.* ^(16)^ and Ohri-Vachaspati *et al.* ^(20)^ highlighted the limitations of categorising non-franchise or non-corporate food retailers with conventions like the North American Industry Classification System (NAICS) used in the mRFEI due to the lack of consensus on inclusion criteria for smaller, heterogeneous retailer types. To improve reliability, some researchers have diverged from coding using NAICS. For instance, while NAICS^(21)^ suggests examining retailer employee numbers to make coding decisions, Ohri-Vachaspati *et al.* opted not to because the possibility of misclassification using this criterion was too great (e.g. convenience stores with many employees could be confused with medium-sized grocery stores)^(20)^.

These concerns are in addition to broader issues with relative RFE measures, such as that relative measures cannot distinguish between RFE with equally low numbers of unhealthy and healthy retailers, and RFE with equally high numbers of unhealthy and healthy retailers – even though these RFE likely influence purchasing and eating practices differently^(12)^. Researchers have suggested that combinations of absolute and relative measures are needed to fully describe RFE^(22,23)^. Indeed, the two Nutrition Report Card indicators discussed herein are interpreted holistically alongside thirty-eight other food environment indicators, upon which recommendations are based.

Grounded in the literature^(10,12,18,20,24)^ and our experiences of developing and implementing spatial accessibility indicators for the Nutrition Report Card, the need for a more comprehensive yet context-specific approach to categorising retailers in RFE assessments was evident. Here, we aim to contribute an operational lens to methodological discussions on measuring RFE.

## Purposes

1. To extend existing RFE measurement strategies by developing and documenting subcategories to increase the granularity of healthy *v*. less healthy options, through inductively coding our Calgary and Edmonton (western Canadian cities) retailer dataset.
2. To establish replicable processes and procedures for coding and updating an RFE database, to increase reliability, avoid miscalculation and reduce the need for manual recategorisation each year.
3. To demonstrate how an RFE codebook and database can be used to generate knowledge mobilisation products (e.g. reports, policy briefs and infographics) for healthy public policy advocacy.

## Methods

### Study setting and population

For the Nutrition Report Card, RFE healthfulness was assessed in Calgary and Edmonton. Calgary is Alberta’s most populous city, with a population of 1 392 609^(25)^. Edmonton, Alberta’s capital, is the second-most populous city with 932 546 people^(26)^. Combined, Calgary and Edmonton account for approximately 60 % of Alberta’s population^(27)^. Calgary and Edmonton have substantial discretion over their RFE through land use planning, informed by the Calgary Food Action Plan^(28)^ and City Environmental Strategies in Edmonton^(29)^.

### Iterative and inductive development of the expanded retailer codebook and database

For each annual Nutrition Report Card, the team collected a complete list of retailers in Alberta from government public health inspectors and used geographic information systems (GIS) software to summarise two spatial accessibility indicators. From this list, we developed a database of retailers categorised as ‘healthy’ and ‘less healthy’ based on CDC mRFEI criteria^(9)^. Our first indicator’s benchmark employed the CDC standard of an average mRFEI score of 10 across all census tracts to our municipalities of interest, indicating a relatively healthful RFE. The Canadian Census defines a census tract as a geographically stable, socio-economically homogenous land area with approximately 4000 residents^(30)^. Our second indicator’s benchmark examined the prevalence of less healthy retailers within 500 m of all public, Catholic, and Francophone Grades K-12 schools in Calgary and Edmonton (addresses obtained from school board websites), characterising the proportion of schools with 0 to 5, *v*. more than 5, retailers within a 500 m radius (see section 2·4). For each iteration of the Nutrition Report Card, our team categorised retailers using the most recent government food inspection list, calculating these two indicators for Calgary and Edmonton.

For the 2015–2019 Nutrition Report Cards, we used the traditional CDC mRFEI criteria^(9)^, adapted to the Canadian context by using the NAICS maintained by Statistics Canada^(21)^. As per mRFEI standards, our analyses were restricted to retailers publicly accessible within the RFE (i.e. retailers within organisational environments like health care facilities and schools were excluded). Less healthy retailers included limited-service eating places (NAICS codes: 722 512_CAN_, 722 211_US_), convenience stores (445 120_CAN&US_), and gas stations with convenience stores (447 110_CAN&US_); healthy retailers included supermarkets and other grocery stores (445 110_CAN&US_), fruit and vegetable markets (445 230_CAN&US_), and general-line food merchant wholesalers (413 110_CAN_, 452 910_US_)^(21)^. For all Nutrition Report Cards (2015–2021), retailers with multiple premise licences were considered a single location and assigned one code, unless a separately licensed premise was a franchise or corporate retailer. For example, a grocery store with separately licensed produce, bakery and meat departments was assigned one overall code (grocery store), while a grocery store with a Starbucks inside was assigned two separate codes (grocery store and limited-service eating place, respectively). This allowed for standardisation between municipalities, as the premise licensing of departments within grocery store locations varied between Calgary and Edmonton.

For the 2020 Nutrition Report Card, we expanded our mRFEI conception. We began by inductively conceptualising the newest version of our retailer codebook and RFE database, developing subcategories with empirical examples that increased the granularity of healthy *v*. less healthy options. Two research team members (human geographer and dietitian with extensive experience updating the dataset) independently created subcategory lists of different retailer types for comparison and consolidation. As in previous years, we started with an initial coding scheme based on the mRFEI. To expand our categorisation, we employed a second column to free code locations which did not correspond to a healthy or less healthy classification (e.g. coffee shops), or which could characterise a possible subcategory for a set classification (e.g. bulk foods stores as a subcategory of supermarkets and other grocery stores). All variations captured across the municipalities were coded this way, with emerging codes and definitions compiled in a codebook by consensus between the two team members. Diagrams were developed to help conceptualise retailer categories and map interrelationships, informed by literature from Daepp and Black^(31)^ and Prowse *et al.* ^(32)^. Each retailer subcategory was then assigned a label of healthy/less healthy, guided by CDC criteria^(9)^. Final subcategories with corresponding healthfulness codes were reviewed and approved by the dietitian in consultation with a senior supervising dietitian on the research team.

For the expanded mRFEI, our healthy retailer definition was broadened to include grocery stores, supermarkets or hypermarkets (incorporating fruit and vegetable markets here), wholesalers, bulk food stores, butchers or delis, fish and seafood shops, juice or smoothie bars, and fresh and healthy quick-service retailers (like sandwich, salad or sushi restaurants). Our less healthy retailers included fast-food restaurants, convenience stores, coffee shops, dollar stores, pharmacies, bubble tea restaurants, candy stores, frozen dessert restaurants, bakeries and food trucks (at their registered location)^(33)^.

To implement the expanded mRFEI for the 2020 Nutrition Report Card, the same two team members coded all retailers in Calgary and Edmonton. After completing the first round of coding, any coding disagreements were resolved by additional review from a third coder who referenced Google Street View, Google Reviews and retailer websites for further information (e.g. storefront and menu details). We report all final subcategories and their assignments as healthy *v*. less healthy, the definitions for each subcategory, and the relative frequencies of each retailer type across the two municipalities, as well as subcategories that were coded but excluded from mRFEI calculations for the Nutrition Report Card (Table 1). These exclusions may support future research on expanding RFE measurement approaches.

**Table 1:** Codebook for categorising retailers based on healthfulness with percent and number of total coded locations†

Category	Description	%	*n*
Healthy			
Sandwich or sushi restaurant	A fast, casual restaurant offering healthier meal items, like sandwiches, soups, salads, sushi, or poke, prepared to order for immediate on-premises and off-premises consumption.	5·41	587
Grocery store*, supermarket* or hypermarket	A retail store offering traditional grocery items such as dairy products, grains, baked goods, produce, pantry staples, meats and/or deli foods.	4·50	488
Juice or smoothie bar	A fast, casual restaurant offering juices and smoothies prepared to order for immediate on-premises and off-premises consumption.	0·85	92
Butcher or deli	A retail store primarily selling fresh and processed meats.	0·34	37
Wholesaler*	A retail store offering produce, meat, dairy products, baked goods and other foods in bulk quantities.	0·13	14
Bulk foods store	A retail store primarily engaged in the sale of dry ingredients, like spices, grains, beans and legumes from bulk bins.	0·10	11
Fish and seafood shop	A retail store primarily engaged in the sale of fresh and processed fish and seafoods.	0·10	11
Less healthy			
Fast-food restaurant*	A fast, casual restaurant offering predominantly less healthy meal items high in calories, sodium, fat, and sugar, like burgers, pizza, and fried chicken, which are prepared to order for immediate on-premises and off-premises consumption.	18·04	1958
Convenience store*	A retail store primarily engaged in the sale of pre-packaged snacks and beverages. May be attached to a gas station. Typically open 18 to 24 h per d.	8·95	971
Coffee shop	A fast, casual restaurant primarily engaged in the sale of beverages prepared to order for immediate on-premises and off-premises consumption, often selling snacks and baked goods.	3·81	413
Drugstore	A retail store including a pharmacy that offers snacks or beverages.	3·53	383
Bakery	A retail store primarily engaged in the sale of bread and baked goods.	3·42	371
Dollar store	A retail store offering pre-packaged foods and beverages, in addition to a variety of other goods, often sold at a discount.	1·14	124
Bubble tea restaurant	A fast, casual restaurant predominantly offering bubble teas and a limited menu or meals and desserts, which are prepared to order for immediate on-premises and off-premises consumption.	0·98	106
Confectionery	A retail store primarily engaged in the sale of candy, chocolate and other confectioneries.	0·85	92
Frozen dessert restaurant	A fast, casual restaurant primarily engaged in the sale of ice cream, frozen yogurt, and other desserts for on-premises and off-premises consumption.	0·83	90
Food truck	Freshly prepared and/or highly processed meals available for take-out services at varying locations.	0·18	20
Excluded			
Full-service restaurant	A traditional restaurant offering table service, where eat-in is a more significant portion of sales than takeaway service.	21·70	2355
Specialty foods store	A retail store primarily engaged in pre-packaged lines of specialised foods like cheeses, jams, nuts and/or pickles for home consumption. Goods are retailed via direct sale, mail order or internet shopping.	5·34	580
Night club, pub or bar	A retailer where alcoholic beverages are the predominant item for sale, although foods and other beverages are also sold for on-premises consumption.	3·52	382
Department store	A large retail store offering a variety of goods in different departments. Pre-packaged foods and beverages are available for sale, but they are not a significant proportion of items sold.	2·29	249
Health supplement store	A retail store primarily engaged in retailing health supplement products, such as vitamins, nutrition supplements and body-enhancing supplements, often targeting weight loss.	2·29	249
Recreation facility	A retail store or fast, casual restaurant within a recreation facility offering foods and beverages for on-premises and off-premises consumption.	1·95	212
School	A public or private school kitchen where meals are prepared for on-premises consumption by students and staff.	1·93	210
Entertainment centre	An entertainment centre requiring admission fees, like a movie theatre, where foods and beverages are sold for on-premises consumption.	1·73	188
Hotel, work cafeteria or banquet hall	A hotel, workplace or banquet hall where meals are prepared on-site for on-premises consumption.	2·35	255
Caterer or meal preparation service	A retailer which prepares customisable and group meals for both on-premises and off-premises consumption.	1·05	114
Community or religious centre	A retail store or kitchen within a community or religious centre where foods are sold for on-premises consumption.	1·01	110
Food manufacturer	An establishment where foods and beverages are produced and/or available for distribution.	0·70	76
Cannabis or vape or beer, wine, and liquor store	A retail store primarily engaged in the sale of cannabis, vape or alcohol products, where individually packaged foods and beverages are also available for sale.	0·59	64
Health care facility	A retail store or kitchen located in a health care facility where meals are prepared for on-premises consumption by patients, visitors, and staff.	0·38	41

mRFEI, modified Retail Food Environment Index; NAICS, North American Industry Classification System.

* Retailers coded as these categories were included in calculating the traditional mRFEI.

† Adapted from NAICS^(21)^, Daepp and Black^(31)^, and Ohri-Vachaspati *et al.* ^(20)^

Finally, for the 2021 Nutrition Report Card, we further developed a system of matching previously coded retailers with retailers in subsequent years based on postal codes, retailer names, and addresses, reducing the need for manual re-coding each year. Retailers that renewed their premise licences could be retained with the same categorisation, while those which discontinued operations were removed. New retailers were coded using the same process of double coding and consensus, as described above.

### Expanding the usefulness of existing retail food environment measures using the 2021 dataset

To demonstrate the utility of our retailer codebook and database, we calculated differences between the traditional *v*. expanded mRFEI (see section 2·2) across census tracts in Calgary and Edmonton, using the 2021 dataset. The CDC’s mRFEI is reported as a proportion from 0–100^(9)^:

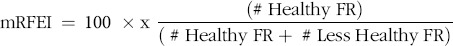




We used descriptive and correlational statistics to test for differences between traditional and expanded mRFEI values, illustrating our comparisons with proximity analysis mapping (see section 2·4 below for details regarding geospatial analysis). For statistical testing, descriptive measures included *total retailers*, *total schools*, *total census tracts* and *total retailers within 500 m of schools* (all dataset-wide totals with information for both Calgary and Edmonton). For our first spatial accessibility indicator measuring RFE across census tracts, we report *total classified retailers*, *total less healthy retailers* and *mean mRFEI* with standard deviation for both the traditional and expanded measures. For our second spatial accessibility indicator measuring RFE in proximity to schools, we report *total classified retailers within 500 m of schools*, *total less healthy retailers within 500 m of schools*, *total schools with less healthy retailers within 500 m*, *mean less healthy retailers within 500 m of schools* and *maximum less healthy retailers within 500 m of schools* for both traditional and expanded mRFEI measures (Table 2). We also calculated Spearman’s rho as a correlation measure to test for differences between the expanded and traditional mRFEI scores obtained across census tracts, and for schools with less healthy retailers within 500 m (Table 2).

**Table 2:** Descriptive and correlational statistics comparing traditional and expanded mRFEI data in Calgary and Edmonton (2021). Values are counts unless otherwise indicated

	Calgary		Edmonton	
Total retailers	6251		4577	
Total schools	367		345	
Total census tracts	227		200	
Total retailers within 500 m of schools	3523		3616	
	Trad.	Exp.	Trad.	Exp.
mRFEI				
Total classified retailers	1488	3188	1355	2533
Total less healthy retailers	1211	2491	1145	2009
mRFEI				
Mean	15	15	13	16
sd	20	17	17	17
Proximity to schools				
Total classified retailers within 500 m of schools	863	1805	1068	2037
Total less healthy retailers within 500 m of schools	688	1449	901	1635
Total schools with less healthy retailers within 50 0m	218	263	235	265
Less healthy retailers within 500 m of schools				
Mean	2	4	3	5
sd	3	6	4	8
Maximum less healthy retailers within 500 m of schools	25	54	40	69
Correlations – Spearman’s rho (*ρ*)				
Census tracts with mRFEI ≥ 10	0·79		0·72	
Schools with less healthy retailers within 500 m	0·89		0·93	

mRFEI, modified Retail Food Environment Index; trad. = traditional mRFEI; Exp. = expanded mRFEI.

### Generating knowledge mobilisation products for healthy public policy advocacy

To illustrate how the retailer codebook and database can be used to generate knowledge mobilisation products, we report geospatial indicators using the expanded mRFEI categorisation for Alberta’s 2021 Nutrition Report Card. The first indicator, measuring whether the benchmark of mRFEI ≥ 10 was met across census tracts in Calgary and Edmonton, was calculated by geocoding all retailer locations and aggregating those points to the census tracts in which they were located, using ArcGIS software^(34)^. For the second indicator, measuring the number of less healthy retailers within 500 m of school locations, we aggregated less healthy retailers to straight-line circular buffers centred on geocoded school locations in each municipality. Notably, for circular buffers intersecting shopping mall locations, we interpreted the value of all retailers within that shopping mall as *falling within* the buffer, approximating unimpeded pedestrian access to multiple locations. Geocoded values from previous years were used in conjunction with new locations geocoded in ArcGIS.

**Fig. 1:**
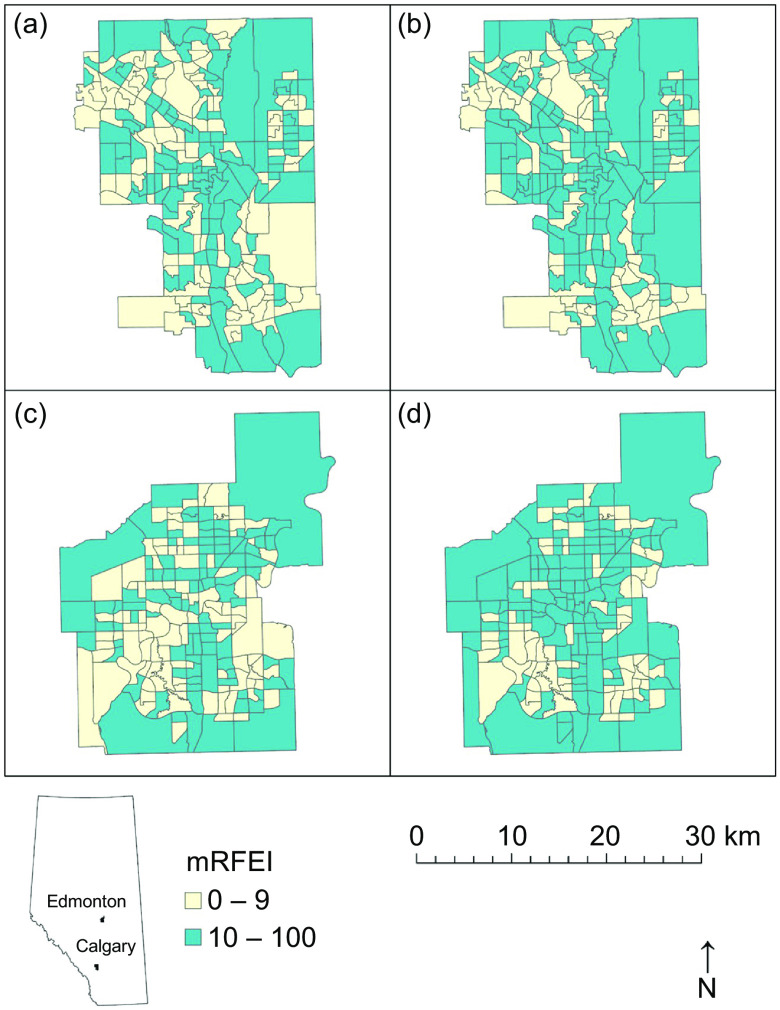
Choropleth maps of the (a) traditional and (b) expanded mRFEI measures for census tracts in Calgary, and (c) traditional and (d) expanded mRFEI measures for census tracts in Edmonton (2021). mRFEI, modified Retail Food Environment Index

**Fig. 2:**
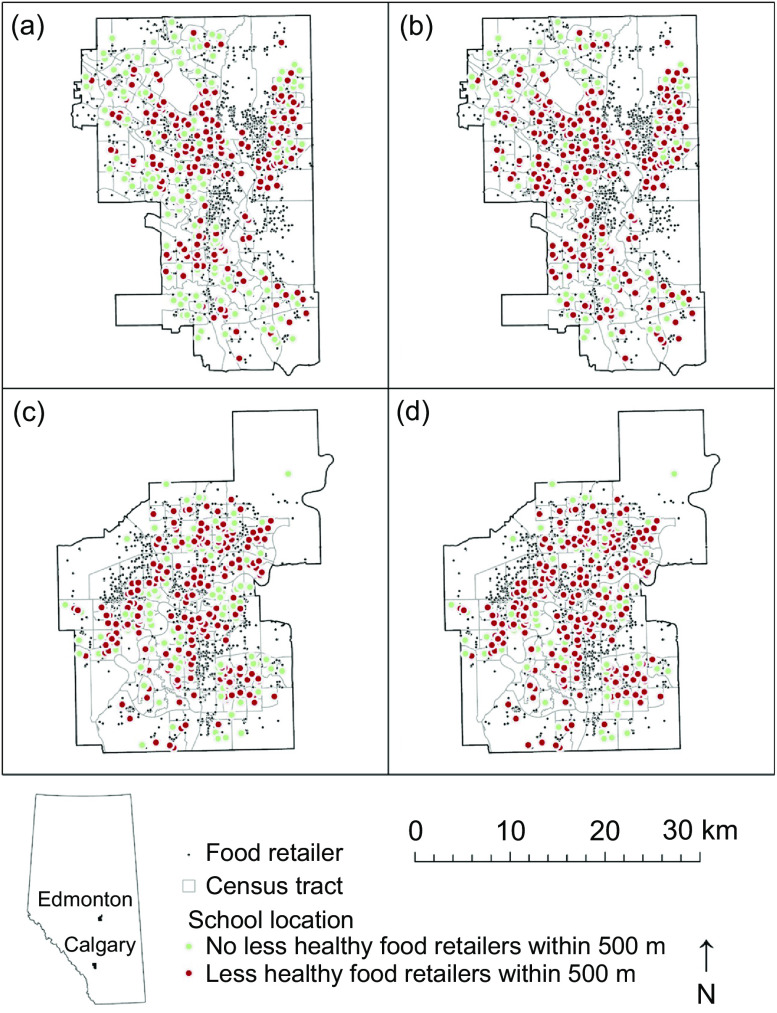
Presence or absence of less healthy retailers within 500 m of schools in Calgary using (a) traditional and (b) expanded mRFEI, and within 500 m of schools in Edmonton using (c) traditional and (d) expanded mRFEI (2021). mRFEI, modified Retail Food Environment Index

**Fig. 3:**
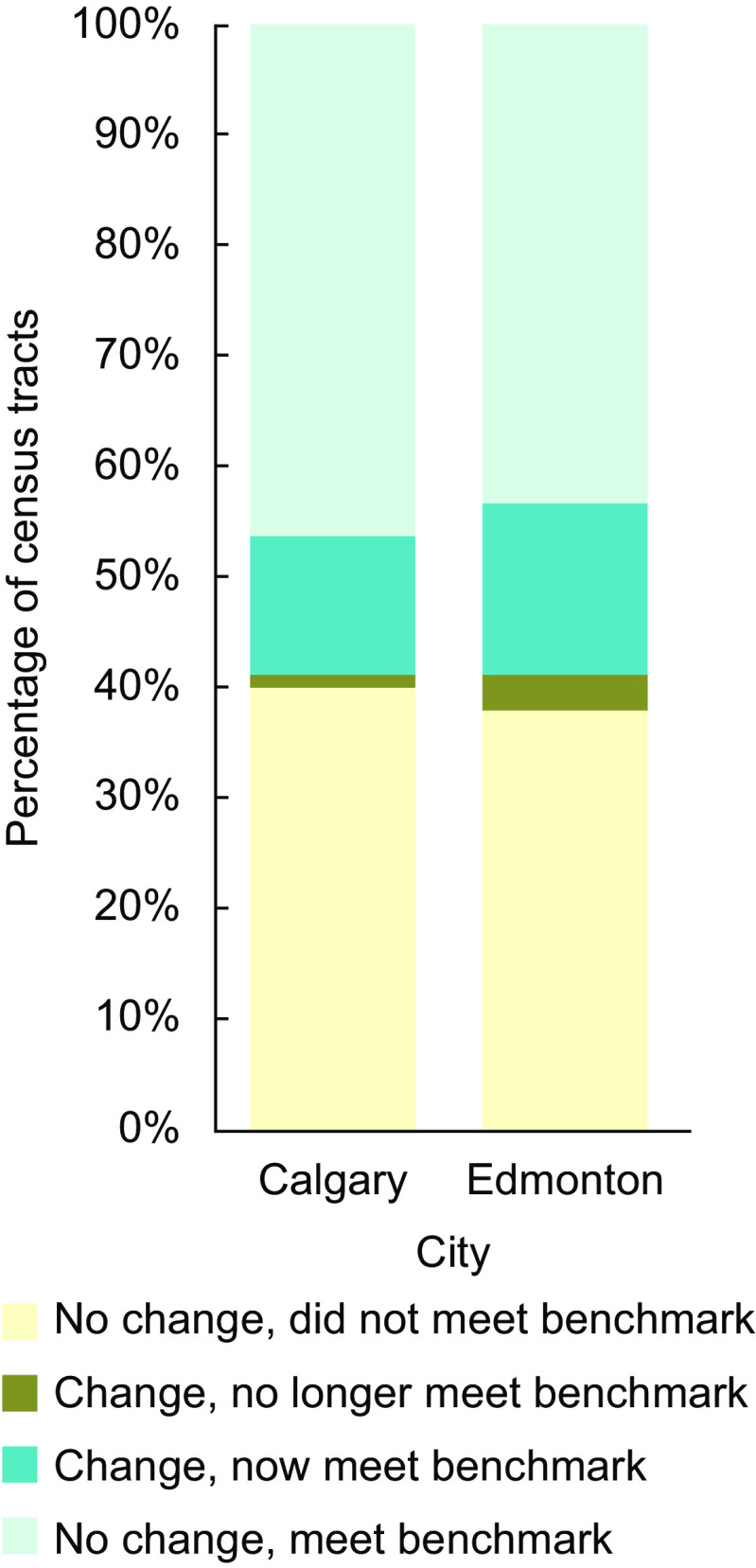
Percentage of census tracts meeting the benchmark of mRFEI ≥ 10 in Calgary and Edmonton, comparing the expanded *v*. traditional measures (2021). mRFEI, modified Retail Food Environment Index

**Fig. 4:**
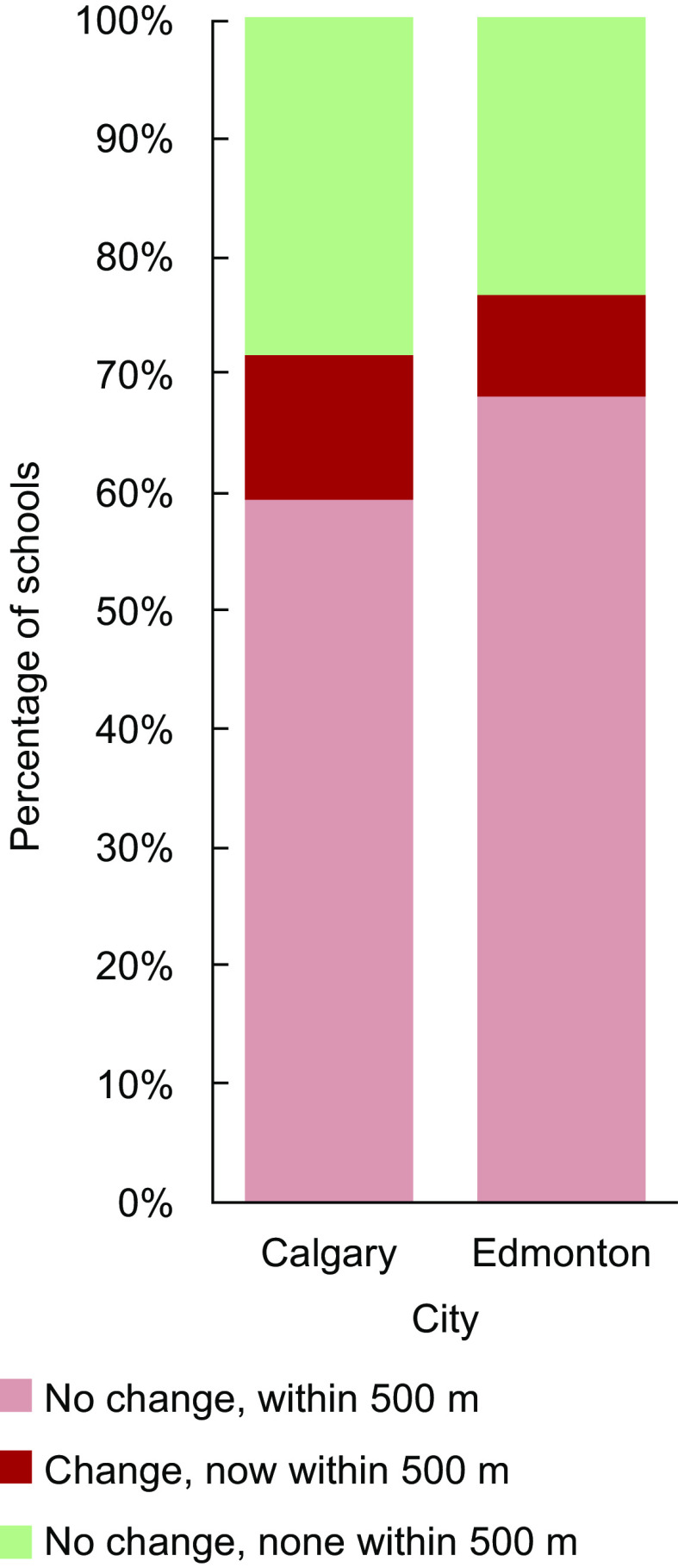
Percentage of schools with one or more less healthy retailers within 500 m, comparing the expanded *v*. the traditional measures (2021)

We mapped mRFEI calculations and the instance of less healthy retailers in proximity to schools, calculating descriptive statistics and correlation with Spearman’s rho using Python 3^(35)^. Thus, we were able to illustrate the spatial distribution of traditional *v*. expanded mRFEI measures for Calgary and Edmonton, mapping both our first (Fig. 1) and second (Fig. 2) indicators. We also report changes in the proportions of census tracts that met the mRFEI ≥ 10 benchmark (Fig. 3) and in the proportion of schools with less healthy retailers located within 500 m (Fig. 4), comparing traditional and expanded mRFEI.

## Results

### Retailer classifications for 2021

There were 10 843 retailers classified with subcategorisations in Calgary and Edmonton, with 10 828 retailers geocoded to census tracts in the 2021 Nutrition Report Card (twenty-five retailers were located on the outskirts of both cities, beyond municipal census tracts)^(33)^. While only 26 % of these geocoded locations would be included using the traditional mRFEI (1488 in Calgary and 1355 in Edmonton), our expanded categorisation enabled us to include 53 % of locations (3188 in Calgary and 2533 in Edmonton). This was achieved by expanding our healthy category to include sandwich/salad/sushi retailers, juice shops, butcher/deli shops, bulk foods and fish shops (in addition to grocery stores and wholesalers) and by expanding our less healthy category to include coffee shops, pharmacies, bakeries, dollar stores, bubble tea shops, confectionery stores, frozen dessert shops and food trucks as less healthy (in addition to fast-food restaurants and convenience stores). Total numbers of subcategorised food retailers prior to geocoding are presented in Table 1, with asterisked categories corresponding to CDC mRFEI categories, albeit with modified nomenclature and definitions. For instance, our fast-food restaurant category corresponds to the CDC-defined limited-service eating place. Our convenience store category encompasses both stand-alone convenience stores and convenience stores attached to gas stations, and our grocery store category incorporates fruit and vegetable markets – despite these being two distinct categories according to the CDC’s NAICS framework.

### Measures and knowledge mobilisation products for 2021

Table 2 presents descriptive and correlational statistics for the mRFEI calculations and proximity analysis. Calgary had 6251 retailers, 367 schools and 227 census tracts; and Edmonton had 4577 retailers, 345 schools and 200 census tracts. The number of classified retailers increased from the traditional to expanded measures for both cities (by 1700 for Calgary and 1178 for Edmonton). Across its census tracts, Calgary’s mean mRFEI remained at 15 for both the traditional and expanded measure, but Edmonton’s mean mRFEI increased from 13 (traditional) to 16 (expanded), indicating slight improvement in overall healthfulness. Both the number of less healthy retailers within 500 m of schools and the number of schools with any less healthy retailers within 500 m significantly increased from the traditional to expanded measures. Using the traditional measures, the average schools in Calgary and Edmonton had two and three less healthy retailers located within 500 m, respectively; this increased to four and five with the expanded measures. In Calgary, the maximum number of less healthy retailers within 500 m increased from 25 (traditional) to 54 (expanded). In Edmonton, the maximum number of less healthy retailer within 500 m increased from 40 (traditional) to 69 (expanded). While census tracts met the mRFEI ≥ 10 benchmark using both the traditional and expanded measures in both cities, the expanded measure captured greater exposure to less healthy retailers in proximity to Calgary and Edmonton schools.

Table 2 also shows correlation statistics for the mRFEI calculations and proximity analysis. Calgary (*ρ* = 0·79) had a slightly higher correlation between traditional and expanded measures when conducting mRFEI calculations than Edmonton (*ρ* = 0·72). Edmonton (*ρ* = 0·93) had a slightly higher correlation between traditional and expanded measures when conducting the 500 m proximity to school analysis than Calgary (*ρ* = 0·89). While high correlation values mean the two measures were similar across census tracts and school locations, there were local differences. Figs 1 and 2 illustrate *where* spatial distribution of those changes occurred; Figs 3 and 4 explain *how* changes occurred (i.e. meet/not meet benchmark or increase/decrease in less healthy retailers).

Differences between the two retailer classification measures are visually depicted in Fig. 1 for the census tract indicator, and Fig. 2 for the proximity to schools indicator, recreating figures developed for Alberta’s 2021 Nutrition Report Card. In Calgary, Fig. 1(a) (traditional) shows that more census tracts met the mRFEI benchmark ≥10 (blue areas) than in Fig. 1(b) (expanded); in Edmonton, a similar pattern is seen in Fig. 1(c) (traditional) and Fig. 1(d) (expanded). Despite greater exposure to less healthy retailers in proximity to schools indicated by the expanded mRFEI measure, the expanded measure nevertheless indicates healthier mean RFE across census tracts in both cities, overall.

Figure 2 shows the prevalence of less healthy retailers within 500 m of schools. In Calgary, there were subtle changes in the number of schools with less healthy retailers within 500 m (red dots) between traditional (Fig. 2 (a)) and expanded (Fig. 2(b)) measures, with the most notable increases along the northwest (44 %; *n* 20) and southwest (27 %; *n* 12). In Edmonton, the change between traditional (Fig. 2(c)) and expanded (Fig. 2(d)) measures is more scattered throughout the city with higher increases in the northwest (43 %; *n* 13) and southwest (23 %; *n* 7). Using the expanded measure to capture more retailer types, Alberta’s 2021 Nutrition Report Card provided key geographic insights about less healthy retailers around schools, enhancing opportunities for knowledge mobilisation to reduce unhealthy RFE exposures for young people.

Figure 3 shows the difference between traditional and expanded mRFEI measures for census tracts. In Calgary, 46 % of census tracts (*n* 105) had no change in terms of meeting a threshold mRFEI value ≥ 10 using either the traditional or expanded measures; 13 % of census tracts (*n* 29) increased their mRFEI to meet the benchmark with the expanded measure; 1 % decreased their mRFEI to not meet it (*n* 2); and 40 % of census tracts (*n* 91) had no change with an mRFEI < 10. In Edmonton, 43 % of census tracts (*n* 86) had no change in meeting an mRFEI ≥ 10 using either of the traditional or expanded measures; 16 % of census tracts (*n* 32) increased their mRFEI to meet the benchmark with the expanded measure; 3 % decreased their mRFEI to not meet it (*n* 6); and 38 % of census tracts (*n* 76) had no change with an mRFEI < 10.

Figure 4 shows how using the expanded measure changed the analysis of retailers within 500 m of schools. In Calgary, the number of schools near less healthy retailers increased from 60 % to 72 % (*n* 218 to 263); the 12 % change (*n* 45) meant there was a decrease from 40 % to 28 % of schools (*n* 149 to 104) *without* less healthy retailers (*P* < 0·001). In Edmonton, the number of schools near less healthy retailers increased from 68 % to 77 % (*n* 235 to 265); the 9 % change (*n* 30) meant there was a decrease from 41 % to 23 % of schools *without* less healthy retailers (*n* 110 to 80) (*P* < 0·001). Put another way, using the expanded measure, there were only 104 schools (or 28·3 % of the total) in Calgary, and 80 schools (or 23·2 % of the total) in Edmonton, that had no less healthy retailers within 500 m. We consider this additional information on the prevalence of less healthy retailers in proximity to schools as the greatest advantage of using the expanded measure for Alberta’s 2021 Nutrition Report Card.

## Discussion

We contribute to RFE measurement literature by fulfilling our objectives of: (1) extending the mRFEI with subcategories capturing additional details about RFE; (2) establishing processes and procedures for redundant coding and ease of annual updating; and (3) demonstrating how the retailer codebook and database were used to generate knowledge mobilisation products for healthy public policy advocacy. Researchers may find value in replicating and tailoring these processes to their local settings.

### Expanded *v*. traditional modified Retail Food Environment Index measures

As described in section 1·3, other researchers have noted incompleteness of traditional RFE measures like the mRFEI^(12,20)^. We provide empirical evidence for these arguments. By using the traditional measure to calculate the proportion of healthy to less healthy retailers in census tracts (as determined from a small list of three healthy and three less healthy retailer types), we illustrated how the mRFEI omits many retailers potentially shaping purchasing and eating practices. Our expanded metric fills these gaps.

When using the expanded *v*. traditional measure for the 2021 dataset, the number of classified retailers increased for both cities; the traditional measure captured 26 % of locations in the list provided by provincial health inspectors, whereas the expanded measure captured more than double the number of these locations with 53 %. Although the mean mRFEI across census tracts in Calgary remained stable, and there was a slight increase in Edmonton, overall, changes in the mRFEI for census tracts were negligible in both cities.

However, when using the expanded mRFEI, the healthfulness of RFE surrounding schools decreased. Less healthy retailers in our expanded measure (coffee shops, pharmacies, bakeries, dollar stores, bubble tea restaurants, confectionery, frozen dessert restaurants and food trucks) were frequently found near schools. Therefore, even if RFE across census tracts remained proportionately stable, RFE around schools may be less healthy than previously thought. Schools are key activity settings for young people; the types of retailers young people are exposed to as they travel between home and their activity settings can affect social norms regarding foods considered ‘common’^(11,36)^. Research has found that retailers geographically accessible to young people, like those surrounding schools, tend to promote energy-dense, nutrient-poor foods and beverages^(11)^. Repeated, compounding exposures to unhealthy RFE may negatively impact young people’s eating practices at a critical point in their development, with eating habits established at this age often extending into adulthood^(37)^. As we focused on the RFE, we did not account for the within-school (i.e. organisational) food environment. But to obtain a fuller picture of environmental influences on children’s eating practices, in-school food environment audits at a representative sample of schools in Calgary and Edmonton could provide valuable contextualising data.

### The importance of context and transparent reporting in geospatial retail food environments analyses

Geospatial analysis is the most common form of RFE measurement^(17)^. Although consolidating and reducing the number of geospatial RFE measurement approaches could enhance comparability across studies, doing so may overlook global RFE variability. Measurement tools developed in one geographical location may not be generalisable to other jurisdictions^(12)^. Measurement validity requires staying true to contextual elements of RFE, like how food norms are established in particular geographic and cultural regions. Considering the need for such variability, transparent reporting is essential^(38)^. Included and excluded retailer types must be reported such that readers can accurately learn from authors’ conclusions. Wilkins *et al.* developed the Geo-FERN (Geographic Information Systems Food Environment ReportiNg) thirty-eight-item checklist which consists of ‘essential’ and ‘desirable’ criteria regarding retailer data sources, data extraction, retailer construct definitions, geocoding methods and access metrics^(18)^. Such a checklist can offer structure and a starting point to normalise comprehensive reporting of RFE measurement methods. It may be useful for researchers intending to apply our expanded measure.

### Limitations and strengths

Our approach addressed some, but not all, mRFEI limitations (see section 1·2). Our measure is intended for high-level use, recognising it may not capture in-store variation in the healthfulness of food and beverage offerings, and that retailer offerings may not directly correlate with what consumers actually purchase and eat^(39)^. To address these broader limitations, researchers have called for multi-level measurement strategies that account for both community (type, availability and accessibility of retailers) and consumer (price, promotion, placement and nutritional quality of food choices) aspects of the RFE^(6,40,41)^. Community food environment measures generally do not account for the accompanying consumer food environment, meaning they may not reflect diet-related exposures of interest. One way to partially address these issues is to conduct in-store/restaurant food environment assessments, using validated tools like the Nutrition Environment Measures Survey for Stores and Restaurants (NEMS-S/R)^(42,43)^, in tandem with geographic access measurements^(10)^. Given our study’s large scale, conducting in-store audits for all 10 843 retailers was not feasible but could be achieved in smaller-scale, community-level RFE assessments.

Geocoding school and retailer locations involved minor manual editing to augment spatial accuracy (e.g. new schools in recent neighbourhood developments). This was important for integrity of the proximity analysis. However, the 500 m distance may not apply to all school types (e.g. distance may not matter for elementary school students who cannot leave schoolgrounds so do not access retailers during the school day but may be greater for high school students who drive). The choice to use census tracts for mRFEI calculations was subject to the modifiable areal unit problem – different values could result from using smaller or larger administrative units when aggregating retailer locations^(44)^. However, census tracts have been consistently used for Alberta’s Nutrition Report Card and are stable units for annual comparisons. Finally, we relied on secondary datasets that were not ground-truthed (a resource-intensive process), which may inaccurately represent spatial accessibility by including retailers that have closed or neglecting some retailers altogether^(31)^.

Strengths of our approach include the ease and rigour of our revised coding process. Having detailed, accurate RFE data (e.g. statistics regarding the number and proportions of specific retailer types) is essential for establishing buy-in among policymakers, to demonstrate how local RFE may not offer people enough healthy eating opportunities^(45)^. The inductive process benefited from being grounded in the team’s years of practical experience applying the coding scheme and our interdisciplinarity (expertise in nutrition, geography and public health). This process was also strengthened by using Google Street View, facilitating deeper knowledge of retailers during desktop coding.

### Future research directions

Our retailer subcategories could be used in at least three additional ways by research, practice and/or policy teams: (1) applying our coding beyond Alberta, since many of the retailer categories in our codebook exist elsewhere in North America; (2) extending the mRFEI by inductively developing unique subcategories based on local data and/or excluded categories outlined in Table 1 (with transparent reporting of codebook details); and (3) developing new geospatial indicators based on indicated and other subcategories. For example, Alberta’s Nutrition Guidelines’^(46)^ healthfulness labels for foods and beverages (choose most often, choose sometimes and choose least often) could be applied to retailer subcategories to assess implementation of jurisdiction-specific nutrition policies, as has been done by team collaborators^(11,31,47)^. Using such guidelines could be one step towards moving past binary categorisations of retailers as healthy/less healthy. Given the increase in less healthy retailers observed using the expanded mRFEI measure in proximity to schools, researchers should continue to problematise young people’s RFE, including RFE around activity settings like recreation centres and childcare facilities^(48)^. Researchers interested in examining adults’ RFE may also benefit from including more retailer types in analyses, like full-service restaurants.

The processes and procedures outlined may be salient for community organisations and education and health authorities keen to catalyse local action for health-promoting change. We are working to translate these coding processes and procedures into an online learning platform and mobile application to guide communities in conducting their own RFE assessments. In doing these assessments, there may be value in examining the potential for retailer misclassification according to neighbourhood demographic composition in Canada, given research findings from Han *et al.* in the USA suggesting that smaller atypical retailers (e.g. convenience stores and specialty food stores) were more likely to be misclassified in Black census tracts than White census tracts^(16)^. The recently released Canadian Food Environment Dataset (Can-FED)^(49)^, which includes nineteen absolute measures and two relative measures (mRFEI and fast-food restaurant mix) of RFE across Canada, offers additional research and collaboration opportunities, such as applying our expanded mRFEI measures to that dataset.

Lastly, our flexible approach allows for adding emergent retailer subcategories over time. This flexibility is important during the COVID-19 pandemic, which has changed how we interact with RFE. For instance, ‘ghost kitchen’ (i.e. delivery-only restaurant with no sit-in space) openings accelerated with lockdowns, and most restaurants now offer delivery^(50)^. Even though delivery is geographically dispersed from physical retailer locations, the normalisation of exposure to these retailers may warrant future investigation.

## Conclusions

This manuscript presents a methodological advancement by novel adaptation of the traditional mRFEI to an expanded mRFEI, which may be valuable for researchers interested in conducting rigorous and comprehensive RFE assessments. We echo others in their calls for more transparent reporting of the processes and procedures used to measure RFE. We argue that increasing the number and precision of retailer subcategories can facilitate a more robust comparison of RFE healthfulness indicators across time and space. Not only does the expanded measure capture exposure to more retailers, but it also allows for nuanced RFE descriptions. Measuring RFE with more specificity can empower communities to improve food environments with interventions tailored to their challenges like zoning bylaws, restrictions on advertising, calorie labelling and other evidence-based tools.
